# Supplementary material for: Genomic Prediction in Local Breeds: The Rendena Cattle as a Case Study
Source: Animals (Basel). 2021 Jun 18;11(6):1815. doi: 10.3390/ani11061815 (PMC8234894; doi:10.3390/ani11061815)
Supplement: Supplementary file 1 [file animals-11-01815-s001.zip › animals-1255463-SI.pdf]

Supplementary Material 1. Value of accuracy, dispersion, and bias divided by the genetic standard deviations (bias_std) for average daily gain. Models presented are Pedigree BLUP (PBLUP), single-step genomic BLUP (ssGBLUP), and different weighting single step, described as follows: non_linear refers to the nonlinear weighting strategies presented in the manuscript with the respective CT value, limit_5 refers to when variance was set up to a maximum of 5, quadratic refers to the quadratic weight applied to the SNP solutions, and sliding stands for the quadratic weight applied to a window of sliding SNPs. iter stands for the number of iterations, and NA values mean that it was not possible to obtain the solution due to a blending problem between $\mathbf{A}^{-1}$ and $\mathbf{G}^{-1}$.

Models	Accuracy	Dispersion	Bias_std
Pblup	0.366	1.140	-0.04
ssGBLUP	0.471	1.046	0.01
non_linear_1.105/iter_1	0.551	1.183	0.00
non_linear_1.105/iter_2	0.552	1.239	0.01
non_linear_1.105/iter_3	0.552	1.245	0.02
non_linear_1.105/iter_4	0.552	1.246	0.02
non_linear_1.105/iter_5	0.552	1.246	0.02
non_linear_1.105/iter_6	0.553	1.246	0.02
non_linear_1.105/iter_7	0.553	1.246	0.02
non_linear_1.105/iter_8	0.553	1.246	0.02
non_linear_1.105/iter_9	0.552	1.246	0.02
non_linear_1.105/iter_10	0.552	1.235	0.02
non_linear_1.105_variance_limit_5/iter_1	0.551	1.183	0.00
non_linear_1.105_variance_limit_5/iter_2	0.552	1.239	0.01
non_linear_1.105_variance_limit_5/iter_3	0.552	1.246	0.02
non_linear_1.105_variance_limit_5/iter_4	0.553	1.247	0.02
non_linear_1.105_variance_limit_5/iter_5	0.553	1.248	0.02
non_linear_1.105_variance_limit_5/iter_6	0.553	1.248	0.02
non_linear_1.105_variance_limit_5/iter_7	0.553	1.249	0.02
non_linear_1.105_variance_limit_5/iter_8	0.553	1.249	0.02
non_linear_1.105_variance_limit_5/iter_9	0.553	1.249	0.02
non_linear_1.105_variance_limit_5/iter_10	0.553	1.249	0.02
non_linear_1.125/iter_1	0.564	1.204	0.00
non_linear_1.125/iter_2	0.567	1.274	0.01
non_linear_1.125/iter_3	0.568	1.283	0.02
non_linear_1.125/iter_4	0.568	1.285	0.02
non_linear_1.125/iter_5	0.568	1.286	0.02
non_linear_1.125/iter_6	0.569	1.286	0.02
non_linear_1.125/iter_7	0.569	1.286	0.02
non_linear_1.125/iter_8	0.568	1.286	0.02
non_linear_1.125/iter_9	0.568	1.286	0.02
non_linear_1.125/iter_10	0.568	1.286	0.02
non_linear_1.125_variance_limit_5/iter_1	0.564	1.204	0.00
non_linear_1.125_variance_limit_5/iter_2	0.568	1.274	0.01
non_linear_1.125_variance_limit_5/iter_3	0.57	1.285	0.02
non_linear_1.125_variance_limit_5/iter_4	0.57	1.289	0.02
non_linear_1.125_variance_limit_5/iter_5	0.571	1.291	0.02
non_linear_1.125_variance_limit_5/iter_6	0.572	1.292	0.02

non_linear_1.125_variance_limit_5/iter_7	0.572	1.294	0.02
non_linear_1.125_variance_limit_5/iter_8	0.573	1.296	0.02
non_linear_1.125_variance_limit_5/iter_9	0.5723	1.296	0.02
non_linear_1.125_variance_limit_5/iter_10	0.573	1.297	0.02
non_linear_1.250/iter_1	0.64	1.303	-0.01
non_linear_1.250/iter_2	0.662	1.466	0.02
non_linear_1.250/iter_3	0.669	1.506	0.02
non_linear_1.250/iter_4	0.67	1.518	0.02
non_linear_1.250/iter_5	0.671	1.523	0.02
non_linear_1.250/iter_6	0.672	1.525	0.02
non_linear_1.250/iter_7	0.673	1.526	0.02
non_linear_1.250/iter_8	0.673	1.527	0.02
non_linear_1.250/iter_9	0.673	1.527	0.02
non_linear_1.250/iter_10	0.673	1.528	0.02
non_linear_1.250_variance_limit_5/iter_1	0.640	1.303	-0.01
non_linear_1.250_variance_limit_5/iter_2	0.668	1.472	0.01
non_linear_1.250_variance_limit_5/iter_3	0.687	1.532	0.02
non_linear_1.250_variance_limit_5/iter_4	0.693	1.563	0.02
non_linear_1.250_variance_limit_5/iter_5	0.693	1.569	0.03
non_linear_1.250_variance_limit_5/iter_6	0.692	1.571	0.03
non_linear_1.250_variance_limit_5/iter_7	0.691	1.568	0.03
non_linear_1.250_variance_limit_5/iter_8	0.690	1.565	0.03
non_linear_1.250_variance_limit_5/iter_9	0.689	1.562	0.03
non_linear_1.250_variance_limit_5/iter_10	0.688	1.561	0.03
quadratic_sliding_variance_20/iter_1	0.472	1.046	0.01
quadratic_sliding_variance_20/iter_2	0.765	1.644	0.02
quadratic_sliding_variance_20/iter_3	NA	NA	NA
quadratic_sliding_variance_50/iter_1	0.472	1.046	0.13
quadratic_sliding_variance_50/iter_2	0.674	1.377	0.38
quadratic_sliding_variance_50/iter_3	0.748	1.318	0.35
quadratic_sliding_variance_150/iter_1	0.472	1.046	0.11
quadratic_sliding_variance_150/iter_2	0.598	1.146	0.31
quadratic_sliding_variance_150/iter_3	0.646	1.032	0.11
quadratic/iter_1	0.472	1.046	0.10
quadratic/iter_2	0.937	1.678	0.61
quadratic/iter_3	0.982	1.438	0.90

Supplementary Material 2. Value of accuracy, dispersion and bias divided by the genetic standard deviations (bias_std) for EUROP. Models presented are pedigree BLUP (PBLUP), single-step genomic BLUP (ssGBLUP), and different weighting single step, described as follows: non_linear refers to the nonlinear weighting strategies presented in the manuscript with the respective CT value, and limit_5 refers to when variance was set up to a maximum of 5. Quadratic refers to the quadratic weight applied to the SNP solutions, and sliding stands for the quadratic weight applied to a window of sliding SNPs. iter stands for the number of iterations, and NA values mean that it was not possible to obtain the solution due to a blending problem between $\mathbf{A}^{-1}$ and $\mathbf{G}^{-1}$.

Models	Accuracy	Dispersion	Bias_std
Pblup	0.509	0.910	-0.009
ssGBLUP	0.597	1.051	0.010
non_linear_1.105/iter_1	0.653	0.959	0.004
non_linear_1.105/iter_2	0.650	0.992	0.010
non_linear_1.105/iter_3	0.650	0.995	0.011
non_linear_1.105/iter_4	0.650	0.996	0.011
non_linear_1.105/iter_5	0.650	0.996	0.011
non_linear_1.105/iter_6	0.650	0.996	0.011
non_linear_1.105/iter_7	0.650	0.996	0.011
non_linear_1.105/iter_8	0.650	0.996	0.011
non_linear_1.105/iter_9	0.650	0.996	0.011
non_linear_1.105/iter_10	0.650	0.996	0.011
non_linear_1.105_variance_limit_5/iter_1	0.653	0.959	0.004
non_linear_1.105_variance_limit_5/iter_2	0.651	0.992	0.010
non_linear_1.105_variance_limit_5/iter_3	0.651	0.997	0.011
non_linear_1.105_variance_limit_5/iter_4	0.651	0.998	0.011
non_linear_1.105_variance_limit_5/iter_5	0.651	0.998	0.011
non_linear_1.105_variance_limit_5/iter_6	0.652	0.999	0.011
non_linear_1.105_variance_limit_5/iter_7	0.652	0.999	0.011
non_linear_1.105_variance_limit_5/iter_8	0.652	1.000	0.011
non_linear_1.105_variance_limit_5/iter_9	0.652	1.000	0.011
non_linear_1.105_variance_limit_5/iter_10	0.652	1.000	0.011
non_linear_1.125/iter_1	0.663	0.968	0.003
non_linear_1.125/iter_2	0.661	1.009	0.010
non_linear_1.125/iter_3	0.661	1.014	0.011
non_linear_1.125/iter_4	0.661	1.015	0.012
non_linear_1.125/iter_5	0.661	1.016	0.012
non_linear_1.125/iter_6	0.661	1.016	0.012
non_linear_1.125/iter_7	0.661	1.016	0.012
non_linear_1.125/iter_8	0.661	1.016	0.012
non_linear_1.125/iter_9	0.661	1.016	0.012
non_linear_1.125/iter_10	0.661	1.016	0.012
non_linear_1.125_variance_limit_5/iter_1	0.663	0.968	0.003
non_linear_1.125_variance_limit_5/iter_2	0.662	1.010	0.010
non_linear_1.125_variance_limit_5/iter_3	0.663	1.017	0.011
non_linear_1.125_variance_limit_5/iter_4	0.663	1.020	0.012
non_linear_1.125_variance_limit_5/iter_5	0.664	1.022	0.012
non_linear_1.125_variance_limit_5/iter_6	0.664	1.023	0.012

non_linear_1.125_variance_limit_5/iter_7	0.665	1.024	0.012
non_linear_1.125_variance_limit_5/iter_8	0.666	1.025	0.012
non_linear_1.125_variance_limit_5/iter_9	0.666	1.026	0.012
non_linear_1.125_variance_limit_5/iter_10	0.666	1.027	0.011
non_linear_1.250/iter_1	0.717	1.012	-0.004
non_linear_1.250/iter_2	0.727	1.105	0.008
non_linear_1.250/iter_3	0.729	1.127	0.012
non_linear_1.250/iter_4	0.730	1.133	0.013
non_linear_1.250/iter_5	0.731	1.135	0.013
non_linear_1.250/iter_6	0.731	1.136	0.014
non_linear_1.250/iter_7	0.731	1.137	0.014
non_linear_1.250/iter_8	0.731	1.137	0.014
non_linear_1.250/iter_9	0.731	1.137	0.014
non_linear_1.250/iter_10	0.732	1.137	0.014
non_linear_1.250_variance_limit_5/iter_1	0.717	1.012	-0.004
non_linear_1.250_variance_limit_5/iter_2	0.733	1.112	0.008
non_linear_1.250_variance_limit_5/iter_3	0.747	1.147	0.010
non_linear_1.250_variance_limit_5/iter_4	0.749	1.162	0.014
non_linear_1.250_variance_limit_5/iter_5	0.749	1.165	0.013
non_linear_1.250_variance_limit_5/iter_6	0.750	1.165	0.014
non_linear_1.250_variance_limit_5/iter_7	0.750	1.165	0.014
non_linear_1.250_variance_limit_5/iter_8	0.749	1.165	0.014
non_linear_1.250_variance_limit_5/iter_9	0.749	1.164	0.014
non_linear_1.250_variance_limit_5/iter_10	0.749	1.164	0.014
quadratic_sliding_variance_20/iter_1	0.575	4.962	0.809
quadratic_sliding_variance_20/iter_2	0.773	6.742	0.845
quadratic_sliding_variance_20/iter_3	0.825	6.665	0.848
quadratic_sliding_variance_50/iter_1	0.575	4.962	0.809
quadratic_sliding_variance_50/iter_2	0.716	6.151	0.836
quadratic_sliding_variance_50/iter_3	0.771	5.675	0.839
quadratic_sliding_variance_150/iter_1	0.575	4.962	0.809
quadratic_sliding_variance_150/iter_2	0.680	5.387	0.849
quadratic_sliding_variance_150/iter_3	0.747	4.796	0.893
quadratic/iter_1	0.575	4.962	0.809
quadratic/iter_2	0.916	7.604	0.888
quadratic/iter_3	0.961	6.975	0.941

Supplementary Material 3. Value of accuracy, dispersion and bias divided by the genetic standard deviations (bias_std) for dressing percentage (DP). Models presented are pedigree BLUP (PBLUP), single-step genomic BLUP (ssGBLUP), and different weighting single step, described as follows: non_linear refers to the nonlinear weighting strategies presented in the manuscript with the respective CT value, limit_5 refers to when variance was set up to a maximum of 5. Quadratic refers to the quadratic weight applied to the SNP solutions, and sliding stands for the quadratic weight applied to a window of sliding SNPs. iter stands for the number of iterations, and NA values mean that it was not possible to obtain the solution due to a blending problem between $\mathbf{A}^{-1}$ and $\mathbf{G}^{-1}$

Models	Accuracy	Dispersion	Bias_std
Pblup	0.463	1.115	-0.002
ssGBLUP	0.528	1.057	0.002
non_linear_1.105/iter_1	0.600	1.156	0.016
non_linear_1.105/iter_2	0.600	1.200	0.022
non_linear_1.105/iter_3	0.600	1.205	0.023
non_linear_1.105/iter_4	0.600	1.206	0.023
non_linear_1.105/iter_5	0.600	1.206	0.023
non_linear_1.105/iter_6	0.600	1.206	0.023
non_linear_1.105/iter_7	0.600	1.206	0.023
non_linear_1.105/iter_8	0.600	1.206	0.023
non_linear_1.105/iter_9	0.600	1.206	0.023
non_linear_1.105/iter_10	0.600	1.206	0.023
non_linear_1.105_variance_limit_5/iter_1	0.600	1.167	0.016
non_linear_1.105_variance_limit_5/iter_2	0.600	1.212	0.022
non_linear_1.105_variance_limit_5/iter_3	0.601	1.218	0.023
non_linear_1.105_variance_limit_5/iter_4	0.602	1.220	0.023
non_linear_1.105_variance_limit_5/iter_5	0.602	1.222	0.023
non_linear_1.105_variance_limit_5/iter_6	0.603	1.223	0.023
non_linear_1.105_variance_limit_5/iter_7	0.602	1.223	0.023
non_linear_1.105_variance_limit_5/iter_8	0.602	1.223	0.023
non_linear_1.105_variance_limit_5/iter_9	0.603	1.223	0.023
non_linear_1.105_variance_limit_5/iter_10	0.603	1.223	0.023
non_linear_1.125/iter_1	0.613	1.173	0.016
non_linear_1.125/iter_2	0.614	1.228	0.022
non_linear_1.125/iter_3	0.614	1.235	0.023
non_linear_1.125/iter_4	0.615	1.237	0.023
non_linear_1.125/iter_5	0.615	1.237	0.023
non_linear_1.125/iter_6	0.615	1.237	0.023
non_linear_1.125/iter_7	0.615	1.238	0.023
non_linear_1.125/iter_8	0.615	1.238	0.023
non_linear_1.125/iter_9	0.615	1.238	0.023
non_linear_1.125/iter_10	0.615	1.238	0.023
non_linear_1.125_variance_limit_5/iter_1	0.613	1.184	0.016
non_linear_1.125_variance_limit_5/iter_2	0.615	1.241	0.022
non_linear_1.125_variance_limit_5/iter_3	0.616	1.250	0.023
non_linear_1.125_variance_limit_5/iter_4	0.618	1.255	0.023
non_linear_1.125_variance_limit_5/iter_5	0.618	1.256	0.023
non_linear_1.125_variance_limit_5/iter_6	0.619	1.257	0.023

non_linear_1.125_variance_limit_5/iter_7	0.619	1.259	0.023
non_linear_1.125_variance_limit_5/iter_8	0.619	1.260	0.023
non_linear_1.125_variance_limit_5/iter_9	0.620	1.260	0.023
non_linear_1.125_variance_limit_5/iter_10	0.620	1.261	0.023
non_linear_1.250/iter_1	0.681	1.252	0.011
non_linear_1.250/iter_2	0.699	1.378	0.022
non_linear_1.250/iter_3	0.703	1.408	0.024
non_linear_1.250/iter_4	0.704	1.416	0.025
non_linear_1.250/iter_5	0.705	1.419	0.025
non_linear_1.250/iter_6	0.706	1.421	0.025
non_linear_1.250/iter_7	0.706	1.421	0.025
non_linear_1.250/iter_8	0.706	1.422	0.025
non_linear_1.250/iter_9	0.706	1.422	0.025
non_linear_1.250/iter_10	0.706	1.422	0.025
non_linear_1.250_variance_limit_5/iter_1	0.681	1.264	0.011
non_linear_1.250_variance_limit_5/iter_2	0.705	1.397	0.021
non_linear_1.250_variance_limit_5/iter_3	0.722	1.443	0.022
non_linear_1.250_variance_limit_5/iter_4	0.727	1.468	0.024
non_linear_1.250_variance_limit_5/iter_5	0.726	1.473	0.026
non_linear_1.250_variance_limit_5/iter_6	0.725	1.472	0.025
non_linear_1.250_variance_limit_5/iter_7	0.724	1.470	0.025
non_linear_1.250_variance_limit_5/iter_8	0.723	1.467	0.025
non_linear_1.250_variance_limit_5/iter_9	0.723	1.466	0.025
non_linear_1.250_variance_limit_5/iter_10	0.723	1.465	0.025
quadratic_sliding_variance_20/iter_1	0.416	5.097	-5.370
quadratic_sliding_variance_20/iter_2	0.494	5.405	-5.432
quadratic_sliding_variance_20/iter_3	0.536	5.910	-4.796
quadratic_sliding_variance_50/iter_1	0.416	5.097	-5.370
quadratic_sliding_variance_50/iter_2	0.458	5.248	-5.107
quadratic_sliding_variance_50/iter_3	0.474	5.302	-4.174
quadratic_sliding_variance_150/iter_1	0.416	5.097	-5.370
quadratic_sliding_variance_150/iter_2	0.459	5.485	-4.698
quadratic_sliding_variance_150/iter_3	0.494	6.073	-4.109
quadratic/iter_1	0.416	5.097	-5.370
quadratic/iter_2	0.673	6.001	-5.533
quadratic/iter_3	0.80	6.495	-5.210
